# Coffee consumption as a double-edged sword for serum lipid profile: findings from NHANES 2005–2020

**DOI:** 10.3389/fnut.2025.1606188

**Published:** 2025-07-09

**Authors:** Chaoyue Mo, Xintong Duan, Junlin Pu, Xuan Zhou, Yongfeng Zheng, Shiyu Wang

**Affiliations:** Chengdu University of Traditional Chinese Medicine, Chengdu, China

**Keywords:** coffee consumption, serum lipid profile, dose-response relationship, National Health and Nutrition Examination Survey, population-based study

## Abstract

**Background:**

There is growing interest in coffee's impact on cardiovascular health. As dyslipidemia is a major modifiable risk factor for cardiovascular disease and coffee may influence lipid metabolism, exploring the association between coffee consumption and serum lipid profile may provide further insights into coffee's cardiovascular effects and help inform dietary recommendations for patients with cardiovascular disease.

**Materials and methods:**

Cross-sectional data were obtained from the National Health and Nutrition Examination Survey (NHANES) 2005–2020. The exposure variable was the daily coffee consumption, measured in cups, and the outcome variables were serum lipid profile components, including total cholesterol (TC), triglycerides (TG), high-density lipoprotein cholesterol (HDL-C), and low-density lipoprotein cholesterol (LDL-C). Weighted multiple linear regression, subgroup and interaction analyses, and restricted cubic spline (RCS) regression were used to evaluate the associations. Sensitivity analyses were conducted to assess the robustness of the results.

**Results:**

Each additional cup of coffee consumed per day was associated with a 1.23 mg/dl increase in TC (95% CI: 0.68, 1.77) and a 1.22 mg/dl increase in LDL-C (95% CI: 0.77, 1.67). In the categorical analysis, among participants consuming ≥3 cups/day, each additional cup of coffee was linked to an increase of 8.45 mg/dl in TC (95% CI: 4.94, 11.96) and 7.86 mg/dl in LDL-C (95% CI: 5.01, 10.72), compared with non-drinkers. In females, HDL-C levels rose with coffee consumption up to 2.6 cups/day, after which they began to decline, showing an inverted U-shaped association. In males, a similar non-linear trend was observed for TG, with levels peaking at 3.0 cups/day before decreasing.

**Conclusion:**

Coffee consumption exerts both beneficial and adverse effects on serum lipid profile. While it is positively associated with elevated TC and LDL-C levels, its relationships with HDL-C and TG are more complex and gender-specific. In females, HDL-C increased with intake up to 2.6 cups/day and declined thereafter, forming an inverted U-shaped pattern. In males, TG followed a similar trend, peaking at 3.0 cups/day. Although these changes were statistically significant, their clinical relevance may vary depending on individual cardiovascular risk profiles.

## 1 Introduction

Coffee is one of the most widely consumed beverages globally, with an estimated 2.25 billion cups consumed daily (1). In the United States, about two-thirds of adults drink coffee every day, accounting for nearly one-fifth of global consumption (2). The impact of coffee consumption on cardiovascular health has attracted increasing attention in recent years (3–6). Serum lipid profile, a key modifiable determinant of cardiovascular risk (7, 8), may be influenced by coffee consumption through various mechanisms, including the actions of bioactive compounds such as diterpenes and modulation of inflammation-related pathways (9–11). Investigating the association between coffee consumption and serum lipid profile may offer further insights into coffee's cardiovascular effects and help inform dietary recommendations for patients with cardiovascular disease.

However, existing research remains controversial. Although some studies have suggested that coffee consumption may raise triglyceride (TG) levels, others have reported the opposite. A meta-analysis of randomized controlled trials reported a significant increase in TG with coffee consumption (12), whereas observational data from the UK Biobank showed an inverse association, with higher consumption linked to lower TG levels (13). Despite the documented positive impact of coffee consumption on high-density lipoprotein cholesterol (HDL-C) in numerous studies (13–15), the evidence remains inconsistent. For example, a decade-long prospective cohort study (16) found that individuals consuming ≥3 cups of coffee per day had significantly lower HDL-C levels compared to non-drinkers (*P* < 0.05). In contrast, no significant association between coffee consumption and HDL-C levels has been reported (17). Additionally, the cholesterol-raising effect of coffee, particularly on total cholesterol (TC) and low-density lipoprotein cholesterol (LDL-C), has been well-documented (13, 18, 19), this effect is primarily attributed to diterpenes such as cafestol and kahweol found in unfiltered coffee (9). Nevertheless, the influence of gender on these associations warrants further investigation.

Besides the inconsistencies regarding the association between coffee consumption and serum lipid profile, limited studies have examined the potential nonlinear patterns of these relationships or explored gender-specific differences using nationally representative data. To address these gaps, our study aimed to evaluate the association between coffee consumption and serum lipid profile using data from a large U.S. adult population, and to investigate whether this association varies by gender.

## 2 Materials and methods

### 2.1 Study participants

This study utilized data from the National Health and Nutrition Examination Survey (NHANES), a nationwide survey employing stratified multistage sampling to evaluate the health and nutrition of the non-institutionalized civilian U.S. population. Ethical approval was granted for the NHANES protocol, and all participants provided written informed consent. The datasets analyzed in this study, along with detailed documentation, are publicly accessible on the NHANES website (20).

**Figure 1:**
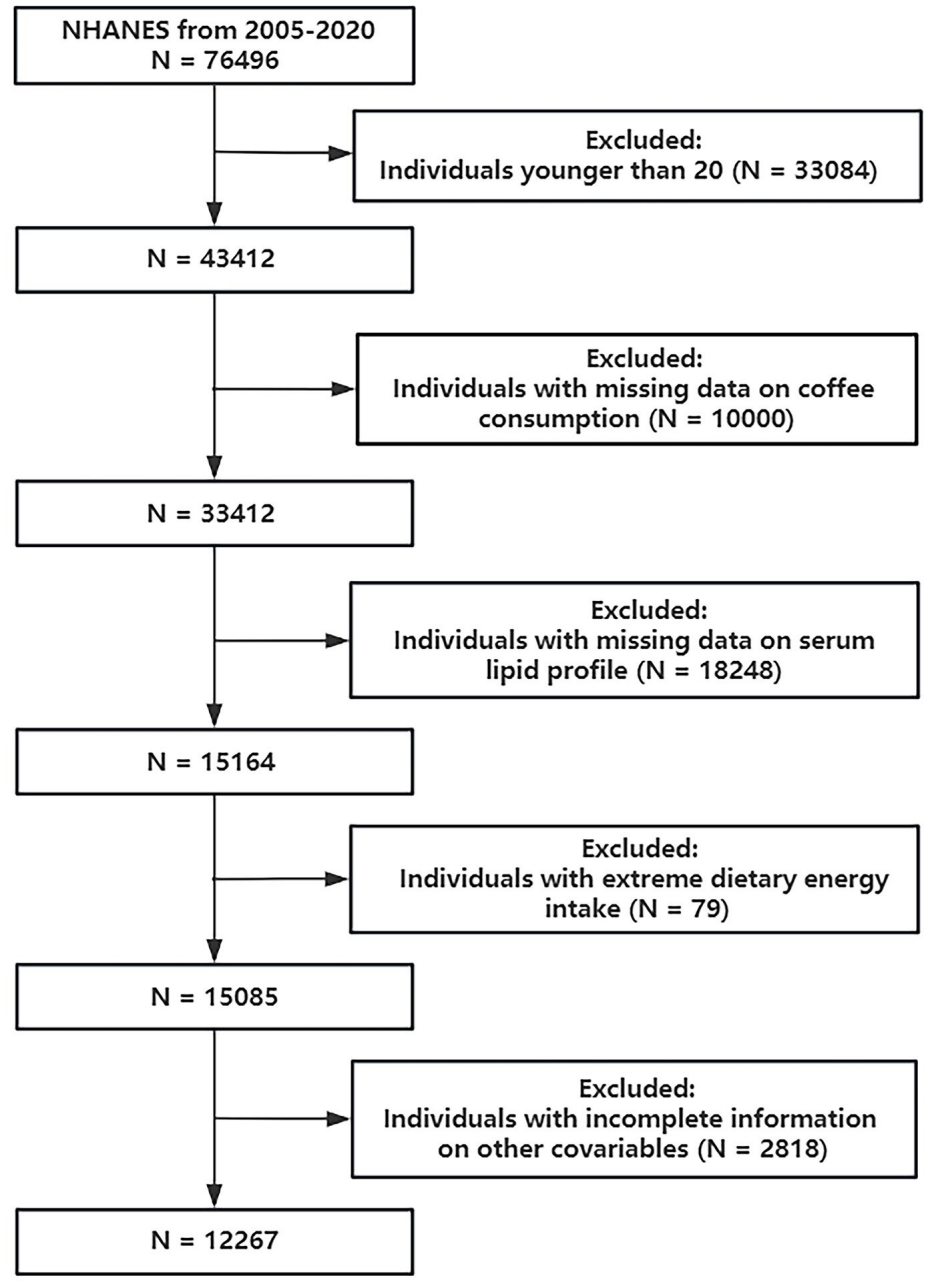
Flowchart of participant selection.

Data from the 2005–2020 cycles of NHANES were employed in this study. Initially, 43,412 subjects aged older than or equal to 20 years were included. Then we excluded individuals according to the following conditions: (1) individuals with missing data on coffee consumption; (2) individuals with missing data on serum lipid profile; (3) individuals with extreme dietary energy intake (<500/>4,000 kcal/day for women and <800/>5,000 kcal/day for men). These thresholds are commonly applied in NHANES-based nutritional epidemiology studies to minimize bias from potential misreporting or measurement errors (21–23); and (4) individuals with incomplete information on other covariates. Finally, 12,267 participants were involved (Figure 1).

### 2.2 Study variables

#### 2.2.1 Assessment of coffee consumption

Coffee consumption was obtained using two 24-h dietary recalls from NHANES 2005–2020, with detailed information derived from the Food and Nutrition Database for Dietary Studies (24). The mean daily consumption from the two recalls was calculated and expressed in cups (1 cup = 283.5 g) (25, 26). Participants were subsequently categorized into four consumption levels: 0, ≤ 1, 1–3, and ≥4 cups per day. These cutoff points were selected based on the distribution of coffee consumption in the study population to ensure adequate statistical power within each group. Similar categorizations have been employed in prior research on coffee consumption and health outcomes (27).

#### 2.2.2 Measurement of serum lipid profile

The serum lipid profile was composed of TC, TG, LDL-C, and HDL-C. TG, HDL-C, and TC were examined via laboratory measurement. LDL-C was calculated from measured values of TC, TG, and HDL-C according to the Friedewald calculation: [LDL-C] = [TC] – [HDL-C] – [TG/5]. The NHANES Laboratory/Medical Technologists Procedures Manual (LPM) covered details about collecting and processing specimens. The commonly accepted normal reference values for lipid profiles in adults are as follows: TC <200 mg/dl, LDL-C <100 mg/dl, HDL-C ≥ 40 mg/dl for men and ≥50 mg/dl for women, and TG <150 mg/dl (28). Participants were classified as having dyslipidemia if they met any of the following conditions: TC ≥ 200 mg/dl, LDL-C ≥ 130 mg/dl, TG ≥ 150 mg/dl, or HDL-C <40 mg/dl in males and <50 mg/dl in females (29).

#### 2.2.3 Covariates

Covariates included age, gender, race, body mass index (BMI), education level, marital status, the ratio of family income to poverty (PIR), physical activity (PA), smoking status, alcohol consumption, energy intake, total sugars intake, dietary fiber intake, total fat intake, total saturated fatty acids intake, cholesterol intake, diabetes, and hypertension. A detailed description of the category variables was provided in Supplementary Table S1.

### 2.3 Statistical analysis

This study adhered to the Centers for Disease Control and Prevention protocols and accounted for sampling weights from the complex, multistage survey design (30). Continuous variables were expressed as mean and standard deviation, while categorical variables were expressed as percentages. All statistical analyses were performed using R software (31), with *P*-value <0.05 considered statistically significant. Three weighted multiple linear regression models were applied to investigate the association between coffee consumption and serum lipid profile. Model 1 was unadjusted. Model 2 adjusted for age, gender, and race. Model 3 adjusted for all covariates. Coffee consumption was modeled both continuously and categorically. Besides, subgroup analysis and interaction test were performed for age, gender, BMI, and smoking status. A Rao–Scott adjusted chi-square test was conducted to examine the association between coffee consumption and dyslipidemia. To assess possible nonlinear trends, restricted cubic spline (RCS) regression was employed. Furthermore, a sensitivity analysis excluding individuals taking lipid-lowering medications was conducted to assess result robustness.

## 3 Results

### 3.1 Baseline characteristics of the study population

Among the 12,267 participants (48.41% male and 51.59% female), the mean age was 47.98 ± 0.33 years. The average serum lipid profile values were: TC, 192.66 ± 0.67 mg/dl; TG, 118.57 ± 1.11 mg/dl; HDL-C, 54.59 ± 0.25 mg/dl; and LDL-C, 114.36 ± 0.52 mg/dl. Of the 12,267 participants, 6,291 (51.28%) were diagnosed with dyslipidemia; 5,810 (47.37%) reported coffee consumption. The proportions of participants who consumed ≤ 1 cup/day, >1 but <3 cups/day, and ≥3 cups/day were 9.55%, 23.83%, and 13.99%, respectively. Among all participants, the average daily coffee consumption was 1.44 ± 0.04 cups. Most of the coffee consumed was caffeinated (1.36 ± 0.04 cups), and the majority of participants preferred adding milk (1.43 ± 0.04 cups), using non-fat options (1.43 ± 0.04 cups), and drinking unsweetened coffee (1.42 ± 0.04 cups). Different levels of coffee consumption show significant differences in age, BMI, PIR, energy intake, total sugars intake, total fat intake, total saturated fatty acids intake, cholesterol intake, TC, TG, HDL-C, LDL-C, gender, race, marital status, education level, PA, smoking status, alcohol consumption, diabetes, and hypertension (all *P* < 0.05; Table 1).

**Table 1:** Weighted baseline characteristics of participants (NHANES 2005–2020).

**Variable**	**Total**	**None**	** ≤ 1 cup/day**	**1–3 cups/day**	**≥3 cups/day**	***P*-value**
*N* (unweighted)	12,267 (100)	6,457 (52.63)	1,172 (9.55)	2,923 (23.83)	1,715 (13.99)	
Age (year)	47.98 ± 0.33	44.63 ± 0.37	46.86 ± 0.78	51.48 ± 0.53	53.13 ± 0.48	**<0.001**
BMI (kg/m^2^)	29.05 ± 0.11	29.33 ± 0.15	29.24 ± 0.30	28.52 ± 0.21	28.90 ± 0.26	**0.005**
PIR	3.05 ± 0.04	2.98 ± 0.05	2.63 ± 0.10	3.14 ± 0.06	3.29 ± 0.07	**<0.001**
Cup of coffee consumption	1.44 ± 0.04	0.00 ± 0.00	0.68 ± 0.01	1.91 ± 0.01	5.14 ± 0.08	**<0.001**
Caffeinated coffee (cup)	1.36 ± 0.04	0.00 ± 0.00	0.61 ± 0.01	1.77 ± 0.02	4.93 ± 0.08	**<0.001**
Coffee with milk (cup)	1.43 ± 0.04	0.00 ± 0.00	0.68 ± 0.01	1.91 ± 0.01	5.14 ± 0.08	**<0.001**
Unsweetened coffee (cup)	1.42 ± 0.04	0.00 ± 0.00	0.67 ± 0.01	1.87 ± 0.02	5.11 ± 0.08	**<0.001**
Fat-free coffee (cup)	1.43 ± 0.04	0.00 ± 0.00	0.68 ± 0.01	1.90 ± 0.02	5.13 ± 0.08	**<0.001**
Energy intake (kcal)	2,106.08 ± 11.84	2,127.63 ± 15.31	1,960.68 ± 37.60	2,023.99 ± 19.37	2,223.68 ± 32.15	**<0.001**
Total sugars intake (g)	110.04 ± 1.10	113.57 ± 1.37	100.66 ± 2.39	105.58 ± 1.50	110.54 ± 2.65	**<0.001**
Dietary fiber intake (g)	17.10 ± 0.18	16.86 ± 0.22	16.62 ± 0.42	17.46 ± 0.26	17.52 ± 0.29	0.064
Total fat intake (g)	81.64 ± 0.56	82.26 ± 0.77	73.07 ± 1.45	78.34 ± 0.98	88.30 ± 1.46	**<0.001**
Total saturated fatty acids intake (g)	26.68 ± 0.20	26.84 ± 0.27	23.51 ± 0.52	25.41 ± 0.34	29.42 ± 0.55	**<0.001**
Cholesterol intake (mg)	289.81 ± 2.55	291.66 ± 3.71	268.86 ± 7.35	275.77 ± 4.42	313.25 ± 5.35	**<0.001**
TC (mg/dl)	192.66 ± 0.67	188.72 ± 0.82	194.38 ± 1.97	194.61 ± 1.17	200.23 ± 1.54	**<0.001**
TG (mg/dl)	118.57 ± 1.11	115.07 ± 1.48	126.88 ± 3.68	120.77 ± 1.99	121.48 ± 2.78	**0.001**
HDL-C (mg/dl)	54.59 ± 0.25	53.72 ± 0.33	54.99 ± 0.69	56.20 ± 0.47	54.66 ± 0.64	**<0.001**
LDL-C (mg/dl)	114.36 ± 0.52	111.98 ± 0.68	114.00 ± 1.53	114.26 ± 0.97	121.26 ± 1.25	**<0.001**
**Gender (%)**						
Female	6,302 (51.59)	3,344 (51.58)	682 (61.66)	1,547 (55.59)	729 (41.59)	**<0.001**
Male	5,965 (48.41)	3,113 (48.42)	490 (38.34)	1,376 (44.41)	986 (58.41)	
**Race (%)**						
Non-Hispanic Black	2,515 (10.00)	1,765 (14.24)	226 (11.10)	413 (6.35)	111 (2.61)	**<0.001**
Mexican American	1,760 (7.70)	845 (8.17)	254 (12.35)	495 (8.34)	166 (3.40)	
Non-Hispanic White	5,698 (70.13)	2,632 (64.59)	383 (57.69)	1,434 (73.39)	1,249 (86.90)	
Other Hispanic	1,112 (5.07)	484 (4.91)	198 (10.02)	321 (5.44)	109 (2.72)	
Other race	1,182 (7.10)	731 (8.09)	111 (8.84)	260 (6.48)	80 (4.37)	
**Marital status (%)**						
Living alone	4,792 (36.46)	2,725 (40.40)	453 (36.37)	1,061 (33.15)	553 (29.97)	**<0.001**
Married or living with partner	7,475 (63.54)	3,732 (59.60)	719 (63.63)	1,862 (66.85)	1,162 (70.03)	
**Education level (%)**						
<High school	2,623 (14.39)	1,212 (13.56)	356 (21.26)	723 (15.37)	332 (12.21)	**<0.001**
≥High school	9,644 (85.61)	5,245 (86.44)	816 (78.74)	2,200 (84.63)	1,383 (87.79)	
**Physical activity (%)**						
No	6,495 (47.71)	3,252 (44.81)	685 (52.78)	1,599 (49.15)	959 (51.48)	**<0.001**
Yes	5,772 (52.29)	3,205 (55.19)	487 (47.22)	1,324 (50.85)	756 (48.52)	
**Smoking status (%)**						
Never	6,754 (54.49)	4,000 (62.81)	721 (59.57)	1,467 (48.58)	566 (36.93)	**<0.001**
Former	3,129 (25.83)	1,305 (19.82)	282 (22.41)	903 (31.24)	639 (36.87)	
Now	2,384 (19.68)	1,152 (17.37)	169 (18.03)	553 (20.17)	510 (26.20)	
**Alcohol consumption (%)**						
Never	1,551 (10.37)	958 (13.43)	189 (11.70)	318 (8.17)	86 (4.20)	**<0.001**
Former	1,946 (13.71)	771 (11.24)	228 (15.39)	570 (14.54)	377 (18.67)	
Mild	4,457 (38.32)	2,309 (36.00)	358 (34.67)	1,117 (43.01)	673 (40.16)	
Moderate	1,948 (17.55)	1,066 (17.31)	170 (17.36)	440 (17.72)	272 (18.10)	
Heavy	2,365 (20.04)	1,353 (22.01)	227 (20.88)	478 (16.56)	307 (18.87)	
**Diabetes (%)**						
No	9,735 (84.28)	5,207 (85.16)	895 (80.23)	2,264 (83.41)	1,369 (84.89)	**0.045**
Yes	2,532 (15.72)	1,250 (14.84)	277 (19.77)	659 (16.59)	346 (15.11)	
**Hypertension (%)**						
No	5,755 (51.89)	3,136 (54.62)	545 (48.82)	1,306 (49.43)	768 (49.03)	**<0.001**
Yes	6,512 (48.11)	3,321 (45.38)	627 (51.18)	1,617 (50.57)	947 (50.97)	

All values are presented as proportion (%) or mean (standard error). Significant values are in bold.

BMI, body mass index; PIR, the ratio of family income to poverty; TC, total cholesterol; TG, triglycerides; HDL-C, high-density lipoprotein cholesterol; LDL-C, low-density lipoprotein cholesterol.

### 3.2 Relationship between coffee consumption and serum lipid profile

In weighted multiple linear regression model 3, when treated as a continuous variable, each additional cup of coffee consumed per day was associated with a 1.23 mg/dl increase in TC (95% CI: 0.68, 1.77) and a 1.22 mg/dl increase in LDL-C (95% CI: 0.77, 1.67). These associations were even more significant when the level of coffee consumption was converted into four groups. For participants with coffee consumption of ≥3 cups/day, each additional cup of coffee was linked to an increase of 8.45 mg/dl in TC (95% CI: 4.94, 11.96) and 7.86 mg/dl in LDL-C (95% CI: 5.01, 10.72), compared with non-drinkers. Although coffee consumption, treated as a continuous variable, showed no significant relationship with TG or HDL-C, the categorical analysis revealed that drinking ≤ 1 cup/day was linked to elevated TG levels (β = 8.63, 95% CI: 2.26, 14.99), while consuming 1–3 cups/day corresponded to higher HDL-C concentrations (β = 1.06, 95% CI: 0.19, 1.92; Table 2).

**Table 2:** Weighted multiple linear regression models for the association between coffee consumption and serum lipid profile.

**Serum lipid profile (mg/dl)**	**Coffee consumption (cups/day)**	β^**a**^ **(95% CI)**, ***P*****-value**		
	**Model 1** ^b^	**Model 2** ^c^	**Model 3** ^d^
TC	Continuous	1.69 (1.16, 2.22) ** <0.001**	1.41 (0.88, 1.94) ** <0.001**	1.23 (0.68, 1.77) ** <0.001**
	None	Reference	Reference	Reference
	≤ 1	5.65 (1.77, 9.54) **0.005**	3.97 (0.10, 7.84) **0.045**	4.26 (0.28, 8.25) **0.036**
	1–3	5.88 (3.25, 8.51) ** <0.001**	3.31 (0.60, 6.01) **0.017**	2.90 (0.30, 5.49) **0.029**
	≥3	11.50 (8.26, 14.75) ** <0.001**	9.6 (6.13, 13.07) ** <0.001**	8.45 (4.94, 11.96) ** <0.001**
	*P* for trend	**<0.001**	**<0.001**	**<0.001**
TG	Continuous	1.00 (0.09, 1.91) **0.031**	−0.24 (−1.14, 0.66) 0.600	−0.41 (−1.27, 0.45) 0.343
	None	Reference	Reference	Reference
	≤ 1	11.81 (4.80, 18.82) **0.001**	10.86 (3.66, 18.06) **0.003**	8.63 (2.26, 14.99) **0.008**
	1–3	5.70 (0.86, 10.54) **0.021**	1.08 (−3.80, 5.95) 0.663	2.23 (−2.38, 6.85) 0.339
	≥3	6.41 (0.00, 12.81) 0.050	−1.28 (−7.79, 5.22) 0.696	−1.25 (−7.64, 5.15) 0.699
	*P* for trend	**0.016**	0.860	0.974
HDL-C	Continuous	0 (−0.21, 0.21) 0.988	0.09 (−0.11, 0.29) 0.385	0.09 (−0.07, 0.25) 0.272
	None	Reference	Reference	Reference
	≤ 1	1.26 (−0.19, 2.71) 0.087	0.36 (−1.02, 1.75) 0.603	0.56 (−0.67, 1.78) 0.370
	1–3	2.48 (1.39, 3.57) ** <0.001**	1.71 (0.66, 2.75) **0.002**	1.06 (0.19, 1.92) **0.017**
	≥3	0.93 (−0.50, 2.37) 0.199	1.36 (−0.06, 2.79) 0.061	0.82 (−0.35, 1.99) 0.167
	*P* for trend	**0.006**	**0.008**	**0.047**
LDL-C	Continuous	1.49 (1.06,1.91) ** <0.001**	1.37 (0.93, 1.80) ** <0.001**	1.22 (0.77, 1.67) ** <0.001**
	None	Reference	Reference	Reference
	≤ 1	2.02 (−1.22, 5.26) 0.220	1.42 (−1.82, 4.67) 0.387	1.97 (−1.37, 5.31) 0.244
	1–3	2.28 (0.09, 4.47) **0.042**	1.40 (−0.84, 3.63) 0.218	1.41 (−0.82, 3.63) 0.213
	≥3	9.28 (6.61, 11.94) ** <0.001**	8.48 (5.67, 11.28) ** <0.001**	7.86 (5.01, 10.72) ** <0.001**
	*P* for trend	**<0.001**	**<0.001**	**<0.001**

In sensitivity analysis, coffee consumption is converted from a continuous variable to a categorical variable; β^a^, effect value; Model 1^b^: no covariates were adjusted; Model 2^c^: adjusted for gender, age, and race; Model 3^d^: adjusted for all covariates. Significant values are in bold.

95% CI, 95% confidence interval; TC, total cholesterol; TG, triglycerides; HDL-C, high-density lipoprotein cholesterol; LDL-C, low-density lipoprotein cholesterol.

**Figure 2:**
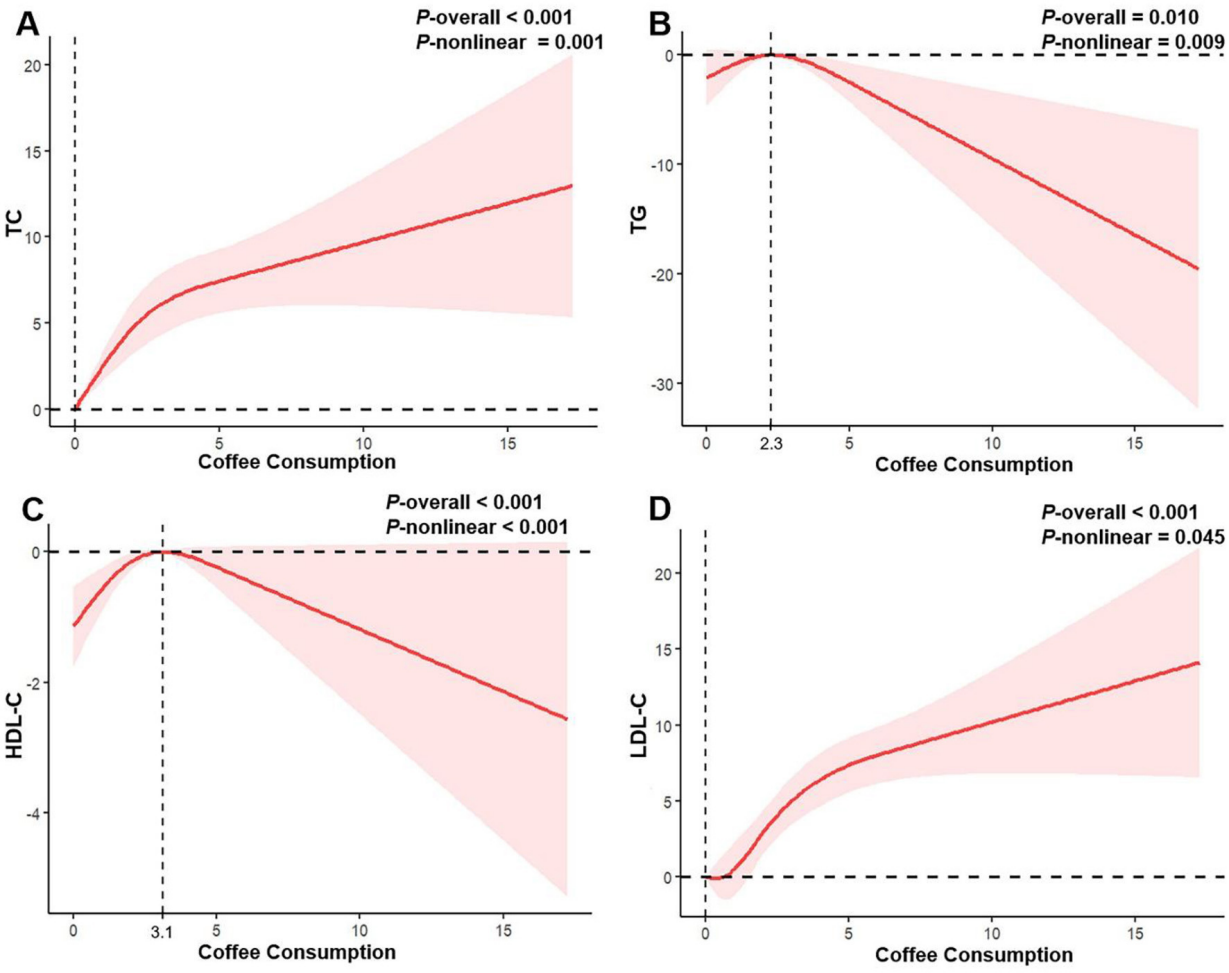
Restricted cubic spline regression analysis of the association between coffee consumption and serum lipid profile in all participants. The solid red lines represent the estimated associations, and the pink shaded regions denote the corresponding 95% confidence intervals. **(A)** Coffee consumption and total cholesterol; **(B)** Coffee consumption and triglycerides; **(C)** Coffee consumption and high-density lipoprotein cholesterol; **(D)** Coffee consumption and low-density lipoprotein cholesterol.

In the RCS (Figure 2), we observed a positive nonlinear relationship between coffee consumption and TC and LDL-C levels (all *P*-nonlinear <0.05), manifested as a rapid increase in initial TC and LDL-C levels with increasing coffee consumption, followed by a slowdown in the rate of increase with further increases in intake. However, no statistically significant inflection point was observed. Interestingly, the associations involving TG and HDL-C appeared to be more complex. Coffee consumption exhibited an inverted U-shaped relationship with TG levels (*P*-nonlinear = 0.009), showing a positive association at intakes below 2.3 cups/day, and an inverse trend beyond this level. Similarly, the initially observed elevation in HDL-C levels with increasing coffee consumption may reverse beyond an intake threshold of 3.1 cups/day (*P*-nonlinear <0.001).

Additionally, there was a significant association between coffee consumption and dyslipidemia status (Rao–Scott adjusted χ^2^ test: *F* = 7.44, df = 2.98, *P* < 0.001).

### 3.3 Gender differences in the relationship between coffee consumption and serum lipid profile

**Figure 3:**
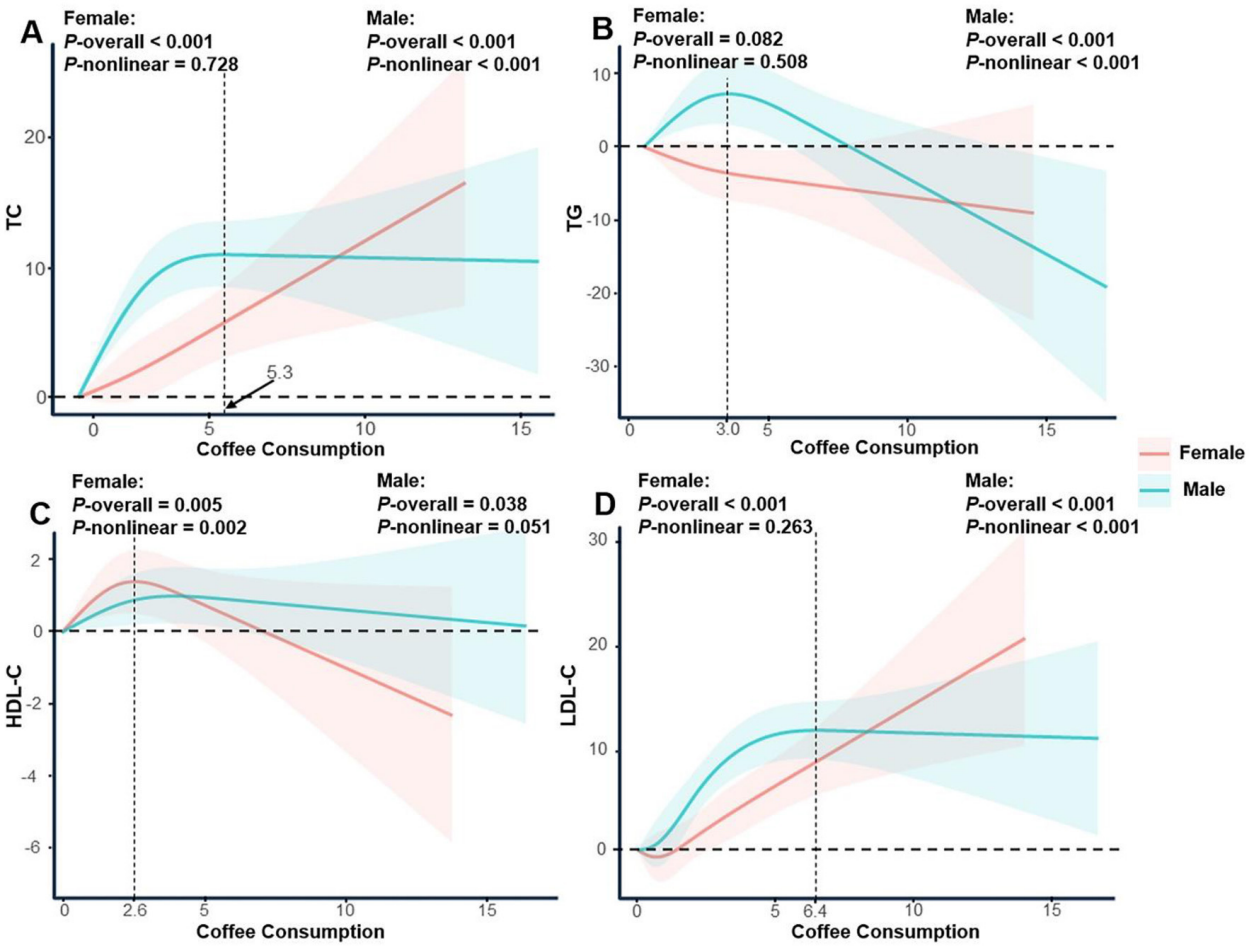
Restricted cubic spline regression analysis of the association between coffee consumption and serum lipid profile stratified by gender. The solid lines represent the estimated associations, and the shaded regions denote the corresponding 95% confidence intervals. **(A)** Coffee consumption and total cholesterol; **(B)** Coffee consumption and triglycerides; **(C)** Coffee consumption and high-density lipoprotein cholesterol; **(D)** Coffee consumption and low-density lipoprotein cholesterol.

In males, coffee consumption exhibited a nonlinear association with both TC and LDL-C levels. TC increased with coffee consumption up to 5.3 cups/day, after which the trend plateaued (*P*-nonlinear <0.001). LDL-C showed a similar trend, with an inflection point at 6.4 cups/day. TG levels in males followed an inverted U-shaped curve, increasing with coffee consumption below 3.0 cups/day and decreasing thereafter. In females, no significant nonlinear associations were observed for TC, LDL-C, or TG (all *P*-nonlinear > 0.05). However, HDL-C levels increased with coffee consumption up to 2.6 cups/day, followed by a downward trend (*P*-nonlinear = 0.002; Figure 3).

### 3.4 Sensitivity analysis

After excluding 2,638 individuals taking lipid-lowering medications, the results suggested that the link between coffee consumption and serum lipid profile remained robust, with the RCS curves exhibiting a consistent direction and shape. In males, there was a nonlinear association between coffee consumption and TC and LDL-C levels. TC increased with increasing coffee consumption up to 5.1 cups/day, after which the trend flattened out (*P* < 0.001). Regarding the effect of coffee consumption on LDL-C levels, although no statistically significant inflection point was observed, the trend aligned with previous analyses, showing that LDL-C initially increased with rising coffee consumption levels before plateauing (*P*-nonlinear <0.001). Male participants' TG levels exhibited an inverted U-shaped curve, increasing when coffee consumption was below 3.3 cups/day and then decreasing. HDL-C also showed a nonlinear relationship, peaking at 3.5 cups/day of coffee consumption and then decreasing. In females, no significant nonlinear associations were observed for TC, LDL-C, or TG (all *P*-nonlinear > 0.05). However, HDL-C levels increased when coffee consumption reached 2.6 cups/day and then showed a decreasing trend (*P*-nonlinear <0.001; Supplementary Table S2 and Supplementary Figures S1, S2).

Overall, the associations between coffee consumption and serum lipid profile, as well as the gender-specific differences, remained robust in the sensitivity analyses. The slight variations in inflection points are likely attributable to differences in sample composition.

### 3.5 Subgroup analysis and interaction test

The results indicated that the association between coffee consumption and serum lipid profile was consistent across subgroups stratified by age, gender, BMI, and smoking status, with no significant interactions observed (all *P* for interaction > 0.05; Supplementary Table S3).

## 4 Discussion

Utilizing data from 12,267 participants in a representative US population sample, we found that coffee consumption was linked to higher levels of TC and LDL-C, yet it might simultaneously have positive influences on TG and HDL-C. These findings suggest that moderate coffee consumption may have a favorable effect on certain lipid components, particularly HDL-C, which is generally considered cardioprotective (32). However, the concurrent rise in TC and LDL-C, known risk factors for atherosclerosis (33), may counteract potential benefits. Therefore, the clinical implications of coffee consumption on lipid metabolism appear to be dose-dependent and require a balanced consideration, especially among individuals at high cardiovascular risk.

Our findings showed that higher coffee consumption was associated with elevated levels of TC and LDL-C, which aligns with previous studies reporting cholesterol-raising effects of coffee (13, 18, 19). This may be largely attributed to the presence of diterpenes such as cafestol and kahweol in unfiltered coffee, which can raise serum cholesterol by reducing hepatic LDL receptor activity and inhibiting bile acid synthesis (9, 10, 34). These findings support the biological plausibility of our results and reinforce concerns about the cholesterol-elevating potential of certain coffee types. Besides, we observed that HDL-C levels increased with coffee consumption up to 3.1 cups/day. Beyond this threshold, further increases in coffee consumption were associated with lower HDL-C levels. This may partly explain the inconsistent findings across the literature. Some studies reported positive associations with HDL-C (14, 35), while others observed adverse effects at higher doses (16, 36). The observed inverted U-shaped curve suggests that moderate coffee consumption may confer lipid benefits, whereas excessive intake may trigger pro-inflammatory responses that counteract these benefits. Zampelas et al. (11) demonstrated a relationship between moderate to high coffee consumption and elevated inflammatory markers, including interleukin-6 (IL-6), C-reactive protein, serum amyloid A, tumor necrosis factor-alpha (TNF-α), and white blood cell count. Given that serum HDL-C levels have been reported to decrease significantly in inflammatory states (37, 38), it is plausible that inflammation serves as a biological mechanism mediating the decline in HDL-C observed with high coffee consumption.

Several previous studies have explored gender-specific associations between coffee consumption and serum lipid profile, and our findings extend this body of evidence. Svatun et al. (39) reported that espresso intake was associated with increased TC, with the effect more pronounced in males. Another large-scale study also showed a significant positive trend between coffee consumption and TC levels among males, whereas no meaningful associations were found in females (40). Our study further revealed that these associations in males tended to plateau at higher consumption levels—specifically, above 5.3 cups/day for TC and 6.4 cups/day for LDL-C—suggesting a dose threshold beyond which the effect may stabilize. In contrast, this plateau was not observed in females. Androgens may contribute to the higher tolerance thresholds observed in males by mitigating inflammation induced by high-dose coffee consumption. One possible explanation is the anti-inflammatory action of testosterone, which has been shown to downregulate pro-inflammatory cytokines—including interferon-gamma, interleukin-2, TNF-α, and IL-6—through suppression of the nuclear factor kappa B pathway, especially via the p65 subunit (41). Additionally, in males, a significant nonlinear relationship resembling an inverted U-curve was observed between coffee consumption and TG levels, whereas no nonlinear association of statistical relevance was detected in females. We hypothesize that coffee may modulate lipocalin-mediated TG metabolism by influencing testosterone levels in males (42, 43). Meanwhile, researchers have proposed that lipocalin mediates the association between coffee consumption and serum triglyceride levels (19). More research is needed to fully elucidate the underlying mechanism.

Our study has several strengths. Weighted multiple regression based on high-quality, nationally representative, large-scale data enhances result credibility, with sensitivity analysis providing additional confirmation of result stability. However, some limitations should be acknowledged. First, due to the cross-sectional nature of our study, causal relationships cannot be established. In particular, reverse causation remains a major concern—individuals with known dyslipidemia may have modified their coffee consumption in response to medical advice or personal health awareness. This behavioral change could obscure the true direction of the association between coffee consumption and serum lipid profile. Second, several coffee-related factors that may impact lipid metabolism—such as brewing method, roast level, and the use of common additives (e.g., cream, sugar)—were not fully considered due to data limitations. Third, the limited number of participants consuming decaffeinated coffee restricted our ability to examine caffeine-independent effects with sufficient statistical power. Future studies with longitudinal designs and more detailed coffee consumption data are warranted to clarify these associations.

## 5 Conclusion

Coffee consumption exerts both beneficial and adverse effects on serum lipid profile. While it is positively associated with elevated TC and LDL-C levels, its relationships with HDL-C and TG are more complex and gender-specific. In females, HDL-C increased with intake up to 2.6 cups/day and declined thereafter, forming an inverted U-shaped pattern. In males, TG followed a similar trend, peaking at 3.0 cups/day. Although these changes were statistically significant, their clinical relevance may vary depending on individual cardiovascular risk profiles.

## Data Availability

The original contributions presented in the study are included in the article/supplementary material, further inquiries can be directed to the corresponding author.
