# Correction: Coffee consumption as a double-edged sword for serum lipid profile: findings from NHANES 2005–2020

**DOI:** 10.3389/fnut.2025.1663658

**Published:** 2025-07-31

**Authors:** Chaoyue Mo, Xintong Duan, Junlin Pu, Xuan Zhou, Yongfeng Zheng, Shiyu Wang

**Affiliations:** Chengdu University of Traditional Chinese Medicine, Chengdu, China

**Keywords:** coffee consumption, serum lipid profile, dose-response relationship, National Health and Nutrition Examination Survey, population-based study

In the published article, there was an error in Figure 2 as published. Figure 2 has been incorrectly replaced with Figure 3. The corrected Figure 2 and its caption appear below.

**Figure 2:**
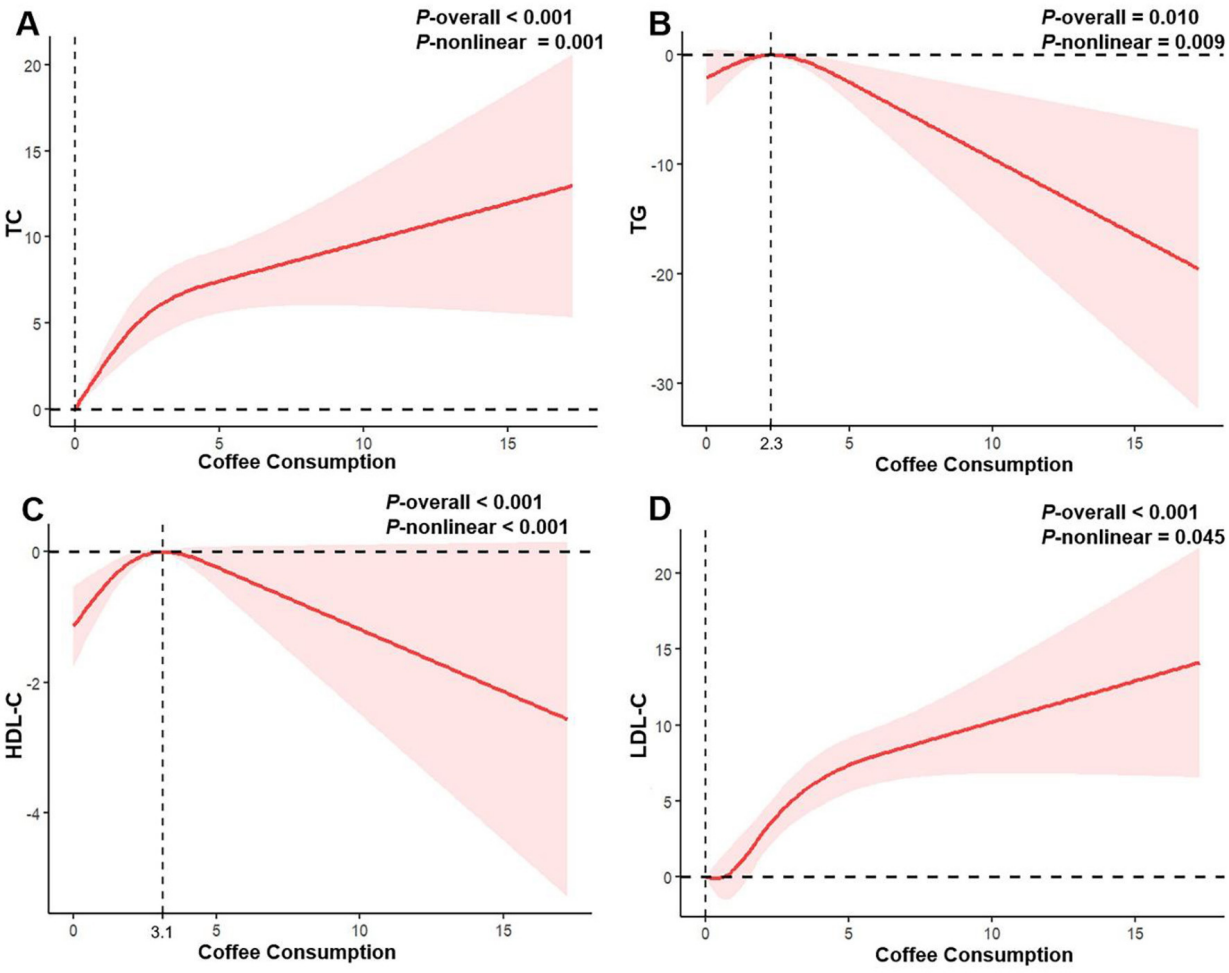
Restricted cubic spline regression analysis of the association between coffee consumption and serum lipid profile in all participants. The solid red lines represent the estimated associations, and the pink shaded regions denote the corresponding 95% confidence intervals. **(A)** Coffee consumption and total cholesterol; **(B)** Coffee consumption and triglycerides; **(C)** Coffee consumption and high-density lipoprotein cholesterol; **(D)** Coffee consumption and low-density lipoprotein cholesterol.

**Figure 3:**
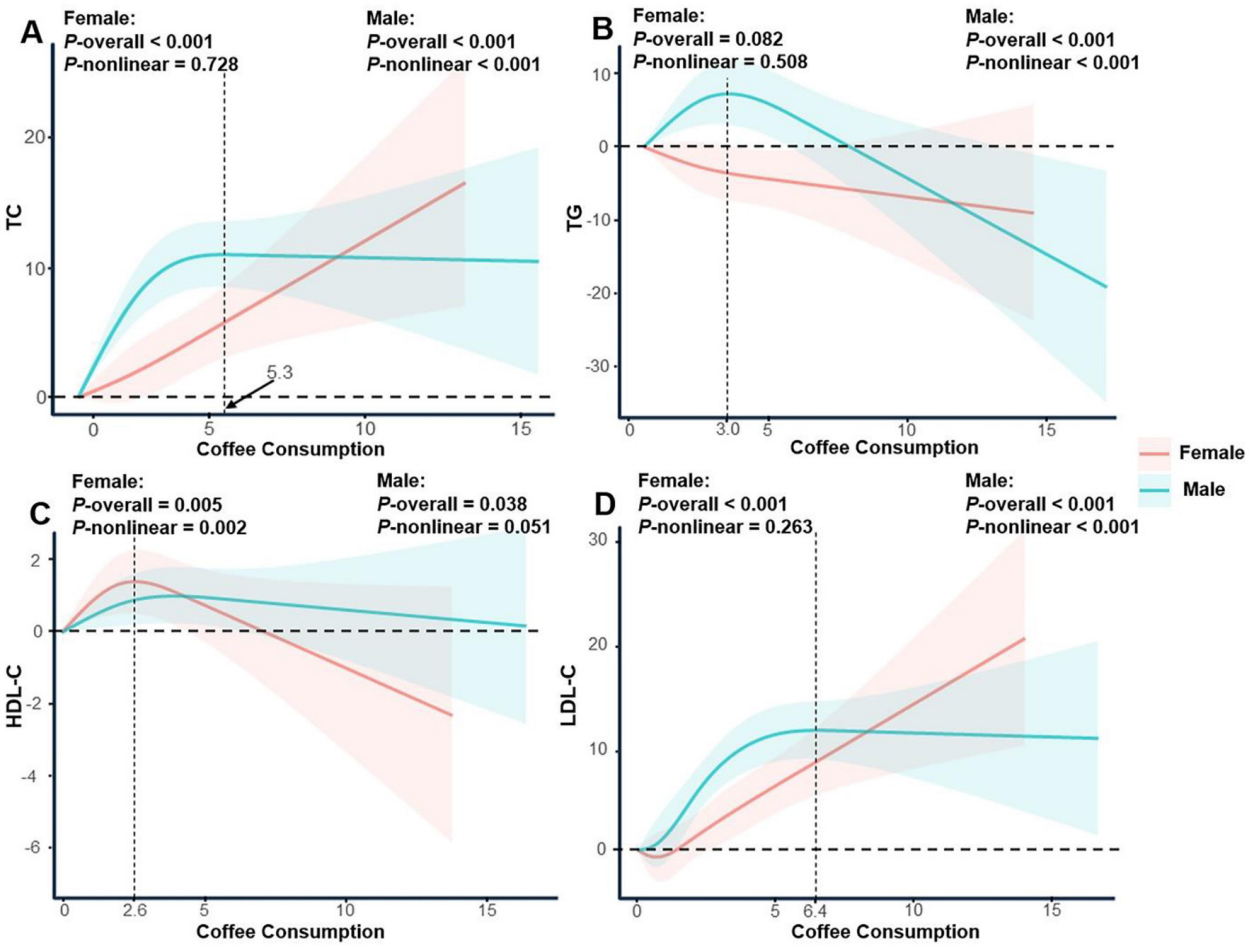
Restricted cubic spline regression analysis of the association between coffee consumption and serum lipid profile stratified by gender. The solid lines represent the estimated associations, and the shaded regions denote the corresponding 95% confidence intervals. **(A)** Coffee consumption and total cholesterol; **(B)** Coffee consumption and triglycerides; **(C)** Coffee consumption and high-density lipoprotein cholesterol; **(D)** Coffee consumption and low-density lipoprotein cholesterol.

In the published article, there was an error in Figure 3 as published. Figure 3 has been incorrectly replaced with Supplementary Figure S1. The corrected Figure 3 and its caption appear below.

The original article has been updated.

