# Supplementary material for: Coffee consumption as a double-edged sword for serum lipid profile: findings from NHANES 2005–2020
Source: Front Nutr. 2025 Jul 9;12:1606188. doi: 10.3389/fnut.2025.1606188 (PMC12283274; doi:10.3389/fnut.2025.1606188)
Supplement: Supplementary file 1 [file Table_1.docx]

Supplementary Material

# Supplementary Figures and Tables

## Supplementary Figures

**
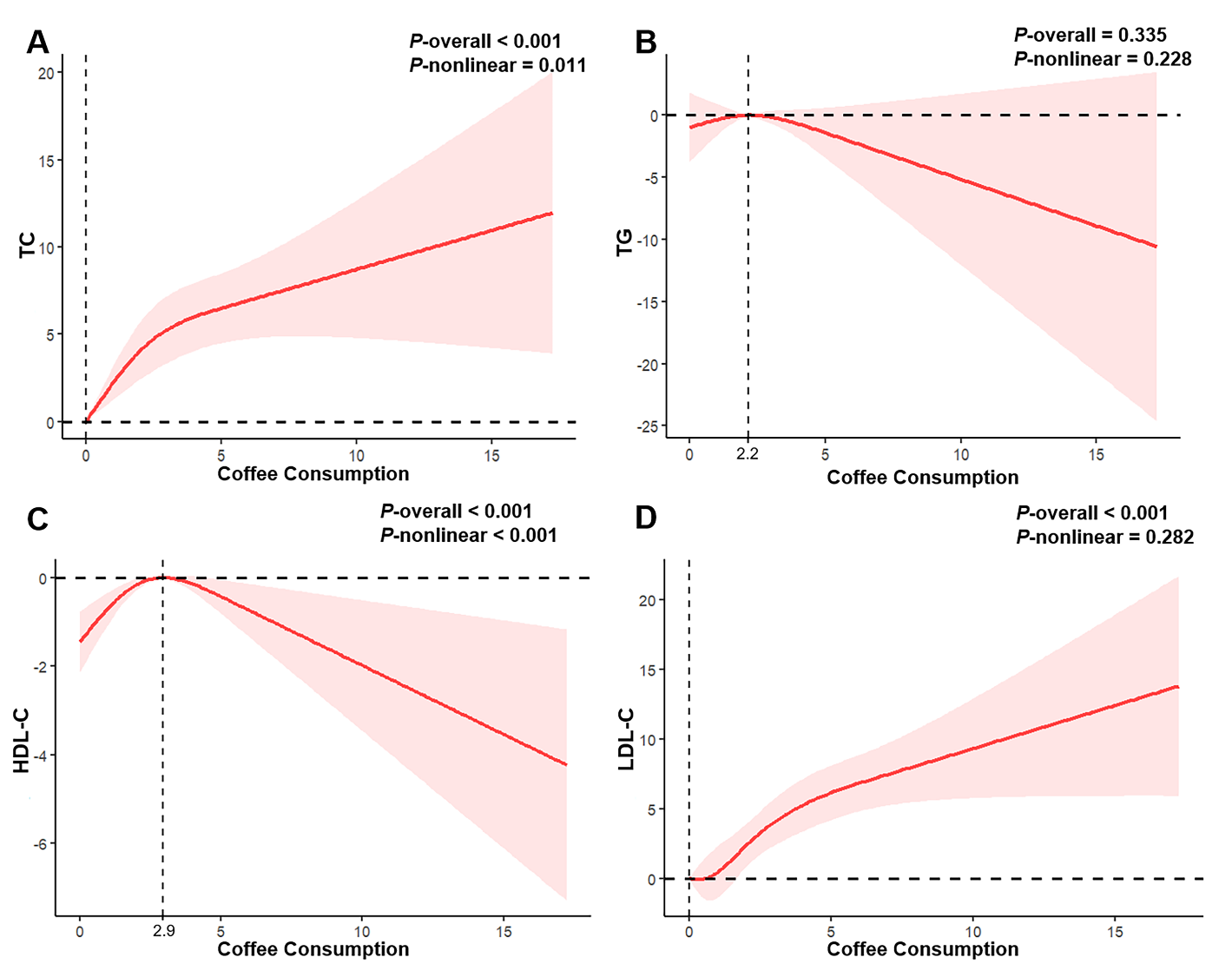
**

**Supplementary Figure S1.** Restricted cubic spline regression analysis of the association between coffee consumption and serum lipid profile in all participants in the sensitivity analysis. The solid red lines represent the estimated associations, and the pink shaded regions denote the corresponding 95% confidence intervals. (A) Coffee consumption and total cholesterol; (B) Coffee consumption and triglycerides; (C) Coffee consumption and high-density lipoprotein cholesterol; (D) Coffee consumption and low-density lipoprotein cholesterol.

**Abbreviations:** TC, total cholesterol; TG, triglycerides; HDL-C, high-density lipoprotein cholesterol; LDL-C, low-density lipoprotein cholesterol

**
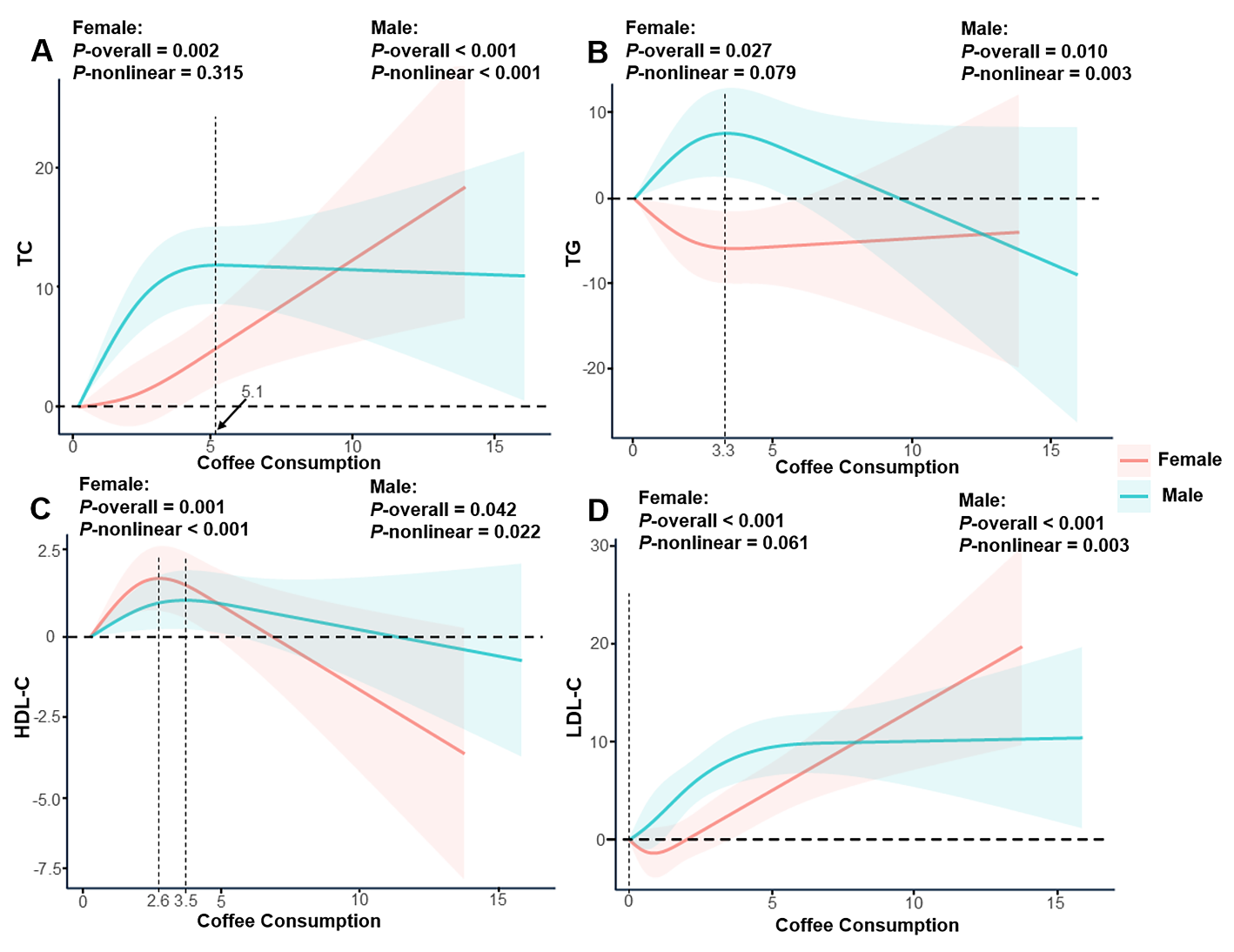
**

**Supplementary Figure S2.** Restricted cubic spline regression analysis of the association between coffee consumption and serum lipid profile stratified by gender in the sensitivity analysis. The solid lines represent the estimated associations, and the shaded regions denote the corresponding 95% confidence intervals. (A) Coffee consumption and total cholesterol; (B) Coffee consumption and triglycerides; (C) Coffee consumption and high-density lipoprotein cholesterol; (D) Coffee consumption and low-density lipoprotein cholesterol.

**Abbreviations:** TC, total cholesterol; TG, triglycerides; HDL-C, high-density lipoprotein cholesterol; LDL-C, low-density lipoprotein cholesterol

- 1. **Supplementary Tables**

**Supplementary Table S1 Detailed information on categorical covariates**

| Covariate | Range | Grouping for adjustment |
| --- | --- | --- |
| Race | Mexican American | Mexican American |
|  | Non-Hispanic White | Non-Hispanic White |
|  | Non-Hispanic Black | Non-Hispanic Black |
|  | Other Race-Including Multi-Racial | Other Race |
| Body mass index (kg/m^2^) | < 18.5 | Underweight |
|  | 18.5-25 | Normal |
|  | 25-30 | Overweight |
|  | ≥ 30 | Obese |
| Education level | Less than 9th grade | < High school |
|  | 9-11th grade (Includes 12th grade with no diploma) | < High school |
|  | High school graduate/GED or equivalent | ≥ High school |
|  | Some college or AA degree | ≥ High school |
|  | College graduate or above | ≥ High school |
| Marital status | Never married | Living alone |
|  | Widowed | Living alone |
|  | Separated | Living alone |
|  | Divorced | Living alone |
|  | Widowed/Divorced/Separated | Living alone |
|  | Living with partner | Married or living with a partner |
|  | Married | Married or living with a partner |
|  | Married/Living with Partner | Married or living with a partner |
| Physical activity | Engaging in any moderate/vigorous recreational activities for at least 10 continuous minutes | Yes |
|  | Participating in muscle-strengthening activities | Yes |
|  | Otherwise | No |
| Smoking status | Has smoked less than 100 cigarettes in their lifetime | Never |
|  | Has smoked more than 100 cigarettes in their lifetime and does not smoke at all now | Former |
|  | Has smoked more than 100 cigarettes in their lifetime and currently smokes some days or every day | Now |
| Alcohol consumption | Had fewer than 12 drinks in their lifetime | Never |
|  | Had ≥ 12 drinks in one year but did not drink last year, or did not drink last year but had ≥ 12 drinks in their lifetime | Former |
|  | Consumed ≤ 1 drink per day for females or ≤ 2 drinks per day for males | Mild |
|  | Consumed ≥ 2 drinks per day for females or ≥ 3 drinks per day for males, or engaged in binge drinking on ≥ 2 days per month, or had a history of daily binge drinking | Moderate |
|  | Consumed ≥ 3 drinks per day for females or ≥ 4 drinks per day for males, or engaged in binge drinking on ≥ 5 days per month | Heavy |
| Diabetes | NA | Diagnostic criteria： |
|  |  | 1. Self-reported physician diagnosis of diabetes 2. Glycated hemoglobin (HbA1c) ≥  6.5% 3. Random blood glucose ≥ 11.1 mmol/L (200 mg/dL) 4. Fasting plasma glucose ≥ 7.0 mmol/L (126 mg/dL) 5. 2-hour plasma glucose ≥ 11.1 mmol/L (200 mg/dL) during an oral glucose tolerance test (OGTT) 6. Current use of insulin or oral glucose-lowering medications |
| Hypertension | NA | Diagnostic criteria： |
|  |  | 1. Self-reported physician diagnosis of hypertension 2. Systolic blood pressure ≥ 130 mmHg and/or diastolic blood pressure ≥ 80 mmHg 3. Current use of antihypertensive medication |
|  |  |  |

**Supplementary Table S2 Weighted multiple linear regression models for the association between coffee consumption and serum lipid profile in the sensitivity analysis**

| **Serum lipid profile**  **(mg/dL)** | **Coffee consumption**  **(cups/day)** | ***β*^a^ (95% CI), *P*-value** | | |
| --- | --- | --- | --- | --- |
|  |  | **Model 1^b^** | **Model 2^c^** | **Model 3^d^** |
| **TC** |  |  |  |  |
|  | **Continuous** | 2.31 (1.66, 2.97) **<0.001** | 1.17 (0.58, 1.76) **<0.001** | 1.08 (0.46, 1.70) **<0.001** |
|  | **None** | **Reference** | **Reference** | **Reference** |
|  | **≤1** | 6.62 (2.54, 10.69) **0.002** | 5.03 (1.19, 8.88) **0.011** | 4.90 (0.99, 8.81) **0.015** |
|  | **1-3** | 7.79 (5.05, 10.52) **<0.001** | 2.36 (-0.47, 5.20) 0.101 | 2.14 (-0.63, 4.90) 0.128 |
|  | **≥3** | 15.66 (11.95, 19.36) **<0.001** | 8.47 (4.75, 12.18) **<0.001** | 7.82 (4.03, 11.61) **<0.001** |
|  | ***P* for trend** | **<0.001** | **<0.001** | **<0.001** |
| **TG** |  |  |  |  |
|  | **Continuous** | 1.13 (0.06, 2.20) **0.038** | -0.15 (-1.21, 0.90) 0.773 | -0.26 (-1.28, 0.76) 0.614 |
|  | **None** | **Reference** | **Reference** | **Reference** |
|  | **≤1** | 11.03 (2.78, 19.29) **0.009** | 10.44 (2.03, 18.85) **0.015** | 8.52 (1.16, 15.87) **0.024** |
|  | **1-3** | 2.91 (-2.06, 7.88) 0.248 | -1.40 (-6.49, 3.68) 0.585 | -0.56 (-5.36, 4.24) 0.817 |
|  | **≥3** | 7.25 (-0.14, 14.64) 0.055 | -0.79 (-8.32, 6.73) 0.835 | 0.01 (-7.43, 7.44) 0.999 |
|  | ***P* for trend** | **0.044** | 0.708 | 0.944 |
| **HDL-C** |  |  |  |  |
|  | **Continuous** | 0.10 (-0.11, 0.32) 0.339 | 0.07 (-0.13, 0.28) 0.496 | 0.07 (-0.10, 0.24) 0.411 |
|  | **None** | **Reference** | **Reference** | **Reference** |
|  | **≤1** | 1.36 (-0.33, 3.05) 0.115 | 0.48 (-1.13, 2.08) 0.555 | 0.49 (-0.89, 1.87) 0.485 |
|  | **1-3** | 3.31 (2.15, 4.46) **<0.001** | 2.15 (1.05, 3.26) **<0.001** | 1.61 (0.70, 2.52) **<0.001** |
|  | **≥3** | 1.54 (0.05, 3.02) **0.043** | 1.29 (-0.24, 2.83) 0.098 | 0.68 (-0.59, 1.95) 0.293 |
|  | ***P* for trend** | **<0.001** | **0.006** | **0.031** |
| **LDL-C** |  |  |  |  |
|  | **Continuous** | 1.98 (1.46, 2.50) **<0.001** | 1.13 (0.65, 1.61) **<0.001** | 1.06 (0.54, 1.57) **<0.001** |
|  | **None** | **Reference** | **Reference** | **Reference** |
|  | **≤1** | 3.03 (-0.44, 6.51) 0.087 | 2.45 (-0.91, 5.81) 0.152 | 2.69 (-0.74, 6.12) 0.123 |
|  | **1-3** | 3.91 (1.62, 6.21) **0.001** | 0.50 (-1.84, 2.84) 0.671 | 0.65 (-1.75, 3.05) 0.591 |
|  | **≥3** | 12.66 (9.63, 15.69) **<0.001** | 7.32 (4.28, 10.35) **<0.001** | 7.12 (3.97,10.28) **<0.001** |
|  | ***P* for trend** | **<0.001** | **<0.001** | **<0.001** |

**Note:** In sensitivity analysis, coffee consumption is converted from a continuous variable to a categorical variable; β^a^, effect value; Model 1^b^: no covariates were adjusted; Model 2^c^: adjusted for gender, age, and race; Model 3^d^: adjusted for all covariates. Significant values are in bold

**Abbreviation:** 95% CI, 95% confidence interval; TC, total cholesterol; TG, triglycerides; HDL-C, high-density lipoprotein cholesterol; LDL-C, low-density lipoprotein cholesterol

**Supplementary Table S3 Subgroup analysis for the association between coffee consumption and serum lipid profile**

| **Serum lipid profile**  **(mg/dL)** | **Subgroup** | ***β*^a^ (95% CI)** | ***P-*value** | ***P* for interaction** |
| --- | --- | --- | --- | --- |
| **TC** |  |  |  |  |
|  | **Age (year)** |  |  | 0.931 |
|  | 20-40 | 0.60 (-0.24, 1.44) | 0.159 |  |
|  | 40-60 | 0.52 (-0.32, 1.37) | 0.224 |  |
|  | ≥60 | 1.52 (0.52, 2.53) | **0.003** |  |
|  | **Gender** |  |  | 0.446 |
|  | Female | 1.14 (0.23, 2.05) | 0.015 |  |
|  | Male | 1.52 (0.77, 2.26) | **<0.001** |  |
|  | **BMI (kg/m^2^)** |  |  | 0.101 |
|  | Underweight | -0.93 (-3.55, 1.69) | 0.405 |  |
|  | Normal | 1.29 (0.17, 2.41) | **0.024** |  |
|  | Overweight | 1.44 (0.38, 2.49) | **0.008** |  |
|  | Obese | 1.06 (0.24, 1.89) | **0.012** |  |
|  | **Smoking status** |  |  | 0.660 |
|  | Never | 0.74 (-0.26, 1.73) | 0.144 |  |
|  | Former | 1.75 (0.71, 2.78) | **0.001** |  |
|  | Now | 1.25 (0.38, 2.13) | **0.006** |  |
| **TG** |  |  |  |  |
|  | **Age (year)** |  |  | 0.403 |
|  | 20-40 | -1.26 (-3.01, 0.50) | 0.159 |  |
|  | 40-60 | -1.43 (-2.73, -0.13) | **0.031** |  |
|  | ≥60 | 0.46 (-0.98, 1.90) | 0.524 |  |
|  | **Gender** |  |  | 0.806 |
|  | Female | -0.85 (-2.17, 0.48) | 0.208 |  |
|  | Male | 0 (-1.20, 1.20) | 1.000 |  |
|  | **BMI (kg/m^2^)** |  |  | 0.144 |
|  | Underweight | -1.52 (-4.55, 1.52) | 0.255 |  |
|  | Normal | 0.30 (-0.80, 1.41) | 0.587 |  |
|  | Overweight | 0.08 (-1.37, 1.54) | 0.910 |  |
|  | Obese | -1.74 (-3.02, -0.45) | **0.009** |  |
|  | **Smoking status** |  |  | 0.638 |
|  | Never | -1.18 (-2.92, 0.56) | 0.182 |  |
|  | Former | -0.04 (-1.56, 1.49) | 0.963 |  |
|  | Now | -0.36 (-1.76, 1.03) | 0.603 |  |
| **HDL-C** |  |  |  |  |
|  | **Age (year)** |  |  | 0.265 |
|  | 20-40 | 0.30 (0.00, 0.59) | **0.048** |  |
|  | 40-60 | 0.16 (-0.09, 0.41) | 0.200 |  |
|  | ≥60 | -0.04 (-0.33, 0.26) | 0.803 |  |
|  | **Gender** |  |  | 0.775 |
|  | Female | 0.1 (-0.20, 0.40) | 0.503 |  |
|  | Male | 0.11 (-0.09, 0.32) | 0.270 |  |
|  | **BMI (kg/m^2^)** |  |  | 0.368 |
|  | Underweight | 0.19 (-0.97, 1.35) | 0.689 |  |
|  | Normal | 0.26 (-0.08, 0.59) | 0.130 |  |
|  | Overweight | 0.07 (-0.19, 0.33) | 0.608 |  |
|  | Obese | 0.18 (-0.08, 0.43) | 0.180 |  |
|  | **Smoking status** |  |  | 0.177 |
|  | Never | 0.29 (0.01, 0.58) | **0.043** |  |
|  | Former | -0.04 (-0.38, 0.30) | 0.837 |  |
|  | Now | 0.12 (-0.12, 0.37) | 0.320 |  |
| **LDL-C** |  |  |  |  |
|  | **Age (year)** |  |  | 0.948 |
|  | 20-40 | 0.55 (-0.20, 1.30) | 0.149 |  |
|  | 40-60 | 0.64 (-0.09, 1.37) | 0.083 |  |
|  | ≥60 | 1.47 (0.55, 2.39) | **0.002** |  |
|  | **Gender** |  |  | 0.485 |
|  | Female | 1.20 (0.40, 2.01) | **0.004** |  |
|  | Male | 1.40 (0.79, 2.01) | **<0.001** |  |
|  | **BMI (kg/m^2^)** |  |  | 0.267 |
|  | Underweight | -0.82 (-3.00, 1.36) | 0.377 |  |
|  | Normal | 0.97 (0.04, 1.90) | **0.040** |  |
|  | Overweight | 1.35 (0.49, 2.21) | **0.002** |  |
|  | Obese | 1.23 (0.49, 1.98) | **0.001** |  |
|  | **Smoking status** |  |  | 0.430 |
|  | Never | 0.68 (-0.11, 1.46) | 0.091 |  |
|  | Former | 1.79 (0.93, 2.64) | **<0.001** |  |
|  | Now | 1.20 (0.40, 1.99) | **0.004** |  |

**Note:** Adjusted for age, gender, race, BMI, education level, marital status, PIR, PA, smoking status, alcohol consumption, energy intake, total sugars intake, dietary fiber intake, total fat intake, total saturated fatty acids intake, cholesterol intake, diabetes, and hypertension except for subgroup variables; β^a^, effect value. Significant values are in bold.

**Abbreviation:** 95% CI, 95% confidence interval; TC, total cholesterol; TG, triglycerides; HDL-C, high-density lipoprotein cholesterol; LDL-C, low-density lipoprotein cholesterol; BMI, body mass index
